# Study on Behind Helmet Blunt Trauma Caused by High-Speed Bullet

**DOI:** 10.1155/2020/2348064

**Published:** 2020-02-18

**Authors:** Zhihua Cai, Xingyuan Huang, Yun Xia, Guibing Li, Zhuangqing Fan

**Affiliations:** ^1^College of Mechanical and Electrical Engineering, Hunan University of Science and Technology, Xiangtan, China; ^2^Daping Hospital, Army Medical University, Chongqing, China

## Abstract

The mechanism of Behind Helmet Blunt Trauma (BHBT) caused by a high-speed bullet is difficult to understand. At present, there is still a lack of corresponding parameters and test methods to evaluate this damage effectively. The purpose of the current study is therefore to investigate the response of the human skull and brain tissue under the loading of a bullet impacting a bullet-proof helmet, with the effects of impact direction, impact speed, and impactor structure being considered. A human brain finite element model which can accurately reconstruct the anatomical structures of the scalp, skull, brain tissue, etc., and can realistically reflect the biomechanical response of the brain under high impact speed was employed in this study. The responses of Back Face Deformation (BFD), brain displacement, skull stress, and dura mater pressure were extracted from simulations as the parameters reflecting BHBT risk, and the relationships between BHBT and bullet-proof equipment structure and performance were also investigated. The simulation results show that the frontal impact of the skull produces the largest amount of BFD, and when the impact directions are from the side, the skull stress is about twice higher than other directions. As the impact velocity increases, BFD, brain displacement, skull stress, and dura mater pressure increase. The brain damage caused by different structural bullet bodies is different under the condition of the same kinetic energy. The skull stress caused by the handgun bullet is the largest. The findings indicate that when a bullet impacts on the bullet-proof helmet, it has a higher probability of causing brain displacement and intracranial high pressure. The research results can provide a reference value for helmet optimization design and antielasticity evaluation and provide the theoretical basis for protection and rescue.

## 1. Introduction

Behind Helmet Blunt Trauma (BHBT) means that when a high-energy bullet and explosive debris impact bullet-proof helmets, the helmet is deformed without penetration and the back of the helmet is exposed to brain force or shock waves transmitted to the brain which cause damage to the brain [1, 2]. Rafaels et al. analyzed more than 6000 cases of bullets and fragments in war, antiterrorism, and peacekeeping and found that more than 70% of injured patients were wearing bullet-proof helmets and other individual protective equipment, of which more than 50% suffered brain injuries [3]. However, the mechanism of BHBT is still not clear. Therefore, understanding the injury mechanism of BHBT and strengthening the protection of the brain are the urgent problems to be solved.

For the study of nonpenetrating injuries in individual equipment, because it is not possible to experiment with living people, cadaveric head experiments, animal experiments, dummy experiments, and digital simulation methods are the main approaches. The cadaver skull is the most similar substitute for living organisms; it can be employed for studies of the biomechanical response and injury mechanism of tissues effectively. However, the sources of fresh and complete cadaver specimens are very limited, the acquisition of sample is very difficult and costly, and there is poor repeatability. The animal experimental model can simulate the response of biological tissues under impact to a certain extent, but there are some differences between the animal and the human body in anatomical structure and tissue response. The finite element method can replace the biomechanical test to a certain extent and study the damage of various parts of the skull and brain. In the previous ten years, Jazi et al. [4], Yang and Dai [5], Pintar et al. [6], and Tse et al. [7] established the human head biomechanical simulation model and used cadaveric experiments for validations; the finite element method was used widely from then on.

In recent years, a large number of scholars have established head finite element models through various methods and approaches. Through the efforts of generations and generations, finite element models with higher precision have been developed on the original simple model, for example, the ULP [8] (University of Louis Pasteur Model) in France, the WSUBIM [9] (Wayne State University Brain Injury Model) in the United States, the KTH [10] (Kungliga Tekniska Hogskolan) in Sweden, the UCDBTM [11] (University College Dublin Brain Trauma) in Ireland, and the GHBMC [12] (Global Human Body Models Consortium). What is more, the NRL-Simpleware head model [13] is already being used for exploring head injury in the military by the U.S. Naval Research Laboratory.

At present, many scholars have conducted research on the parameters of helmet protection. Pintar et al. [6], Rodríguez-Millán et al. [14], and Palta et al. [15], using the combination of experiment and simulation, studied the injury mechanism of helmet protection under the impact of a bullet. Li et al. [16] established and validated the clay-injected human brain simulation model. Tham et al. [17] used light gas guns to conduct the experiments and simulations. Hai et al. [18] used the landrace and the physical model to study the brain injury caused by different bullet speeds. Rafaels et al. [3] carried out the cadaver experiment of the brain injury caused by the bullet impacting a bullet-proof helmet; Palomar et al. [19] used the finite element method to study the injury of the skull caused by different bullet speeds. What is more, there are also studies on other parameters. For example, Yang and Dai [5] established a human brain biomechanical simulation model and carried out simulation analysis of brain damage caused by the bullet impact at different angles and different positions. Tan et al. [20] researched on both experiments and numerical simulations of frontal and lateral ballistic impacts on the Hybrid III headform equipped with Advanced Combat Helmets (ACH). The results show that a finite element model of human brain biomechanics with high fidelity can accurately simulate the biomechanical response and damage of the brain under the impact of a bullet. Jazi et al. [4] established a human brain simulation model to study the biomechanical response of the brain when the bullet impacts the bullet-proof helmet. The parameters of different brain cushions, different impact angles, and different impact positions on the brain were analyzed. Aare and Kleiven [21] used the brain finite element model to simulate the biomechanical response of the brain under the parameters of helmet stiffness and different impact angles.

The purpose of current study is to investigate the response of the human skull and brain tissue under the loading of a bullet impacting the helmet, with the effects of impact direction, speed, and impactor structure being considered. To achieve this, a human brain finite element model which can accurately reconstruct the anatomical structures of the scalp, skull, brain tissue, etc., and can realistically reflect the biomechanical response of the bullet-proof-protected brain under high impact speed was used in this study.

The responses of BFD, brain displacement, skull stress, and dura mater pressure were extracted from simulations as parameters to reflect BHBT risk; the relationships between BHBT and bullet-proof equipment structure and performance were also investigated. The findings can provide a better understanding on the biomechanical response of the brain under the loading of a bullet impacting a bullet-proof helmet.

## 2. Materials and Methods

### 2.1. Establishment of Finite Element Models

**Figure 1:**
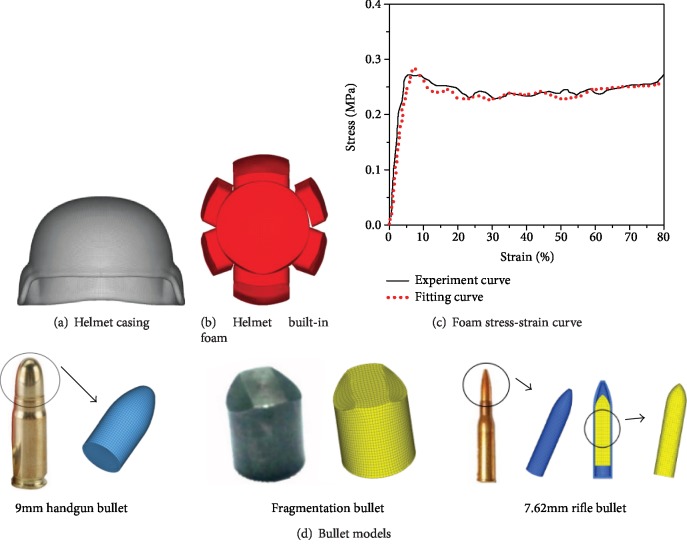
Bullet-proof helmet model and bullet model.

The helmet uses the established and verified US army advanced combat helmet [22] which is shown in Figure 1(a). The helmet model used for this study has 7 foams. The foam models are shown in Figure 1(b). The size of the cylindrical foam is 150 × 30 mm in diameter and height. The length, width, and height of the rectangular blocks are 90∗55∗30 mm and 90∗85∗30 mm; the element size is 3 mm. The advanced combat helmet liner is a polyurethane foam material; the gasket material is a hard foam with stress-strain behavior with load rate sensitivity. According to the properties of the rigid foam material, the MAT_LOW_DENSITY_FOAM (MAT_57) is used in the LS-DYNA material library. The material density is 6.1 × 10^−8^ kg/mm^3^, the Young's modulus is 8.4 MPa, and the relationship between stress and strain was taken from a uniaxial tensile test, and the stress-strain curve which is shown in Figure 1(c) is fixed by MATLAB.

The handgun, fragmentation, and rifle bullets are all constructed by the software PROE (Parametric Technology Corporation, Massachusetts, United States) to create a geometric model. The eight-node hexahedral element is used for meshing in the finite element preprocessing software HyperMesh (Altair Engineering Inc., Troy, MI, USA). The element size of the handgun bullet is 2 mm [20]. The whole model contains 21616 nodes and 1695 elements, and the mass is 8 g. The finite element model of fragmentation [23] is using AISI 4340 steel. The whole model contains 14283 nodes and 12584 elements with a mass of 1.13 g. The rifle bullet finite element model [24] is using a 7.62 mm rifle projectile whose warhead is divided into three parts: armor, steel core, and lead core. The whole model contains 8640 nodes and 5389 elements with a mass of 10.98 g. The finite element model diagrams are shown in Figure 1(d). The bullet material parameters are shown in Table 1.

The established finite element model of the brain [25, 26] is improved, the skin and the skull have surface-to-surface contact, and other brain tissues are using a self-contact algorithm. The cerebrospinal fluid was modified to be an ALE mesh during the preprocessing, the material was changed to ∗MAT_ELADTIC_PLUID (MAT_1), and the unit algorithm was ELFORM = 12. The keyword ∗CONSTRAINED_LAGRANGE_IN_ SOLID is set to achieve the coupling between the structure and the fluid, so that the “skull-cerebrospinal fluid-brain” has a fluid-solid coupling relationship.

### 2.2. Verification of Finite Element Models

The brain pressures and skull responses of the brain finite element model have been verified based on the relevant literature of Cai et al. [25]. This study involved brain displacements, so the brain-skull relative displacements were verified by the brain finite element model with reference to the Hardy et al. experiment [27].

**Figure 2:**
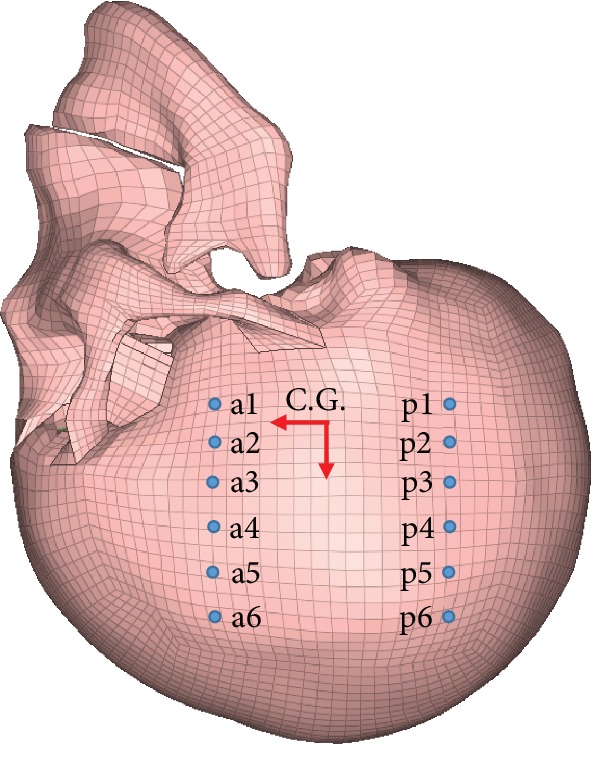
Schematic diagram of head centroid and nodes.

**Figure 3:**
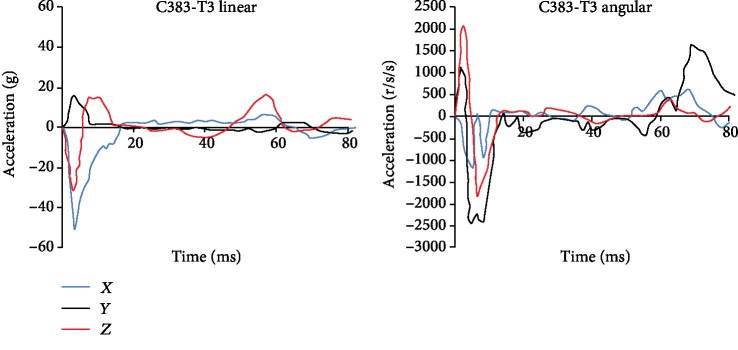
The linear and angular accelerations of C383-T3 [27].

The C383-T3 high-intensity frontal impact test was used to evaluate the relative displacement of the brain. Invert the head model and select the nodes which are close to the position of the NDT identifier in the Hardy et al. experiment. The nodes a1-a6 are the identification of the anterior humerus to the parietal region and p1-p6 are the posterior occiput to the parietal region; the distance of each marker is approximately 10 mm. The head center of gravity which is automatically calculated by HyperMesh and the node positions are shown in Figure 2; the relative displacement of each node and the skull is calculated.  Figure 3 shows the translational acceleration and rotational acceleration measured by the Hardy et al. experiment at the head center of gravity and used as loading conditions on the skull which was modeled as rigid.

**Figure 4:**
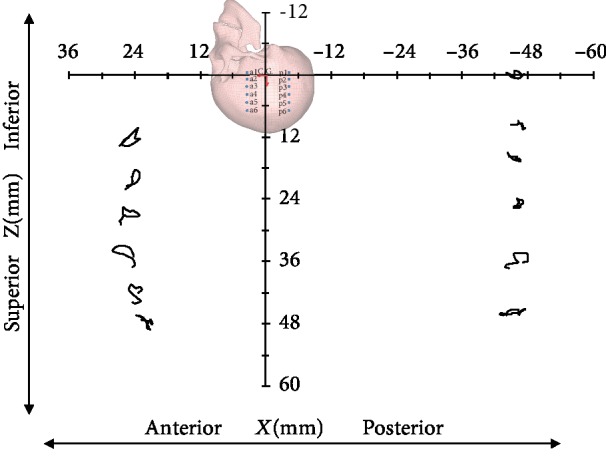
Simulation of brain skull relative displacements.

The 80 ms head motion response process is simulated, and the relative displacement trajectories of the brain are shown in Figure 4. Overall, the selected nodes in the simulation are basically ring-shaped with respect to the movement of the skull during the impact process, which is consistent with the experimentally obtained motion trajectory.

**Figure 5:**
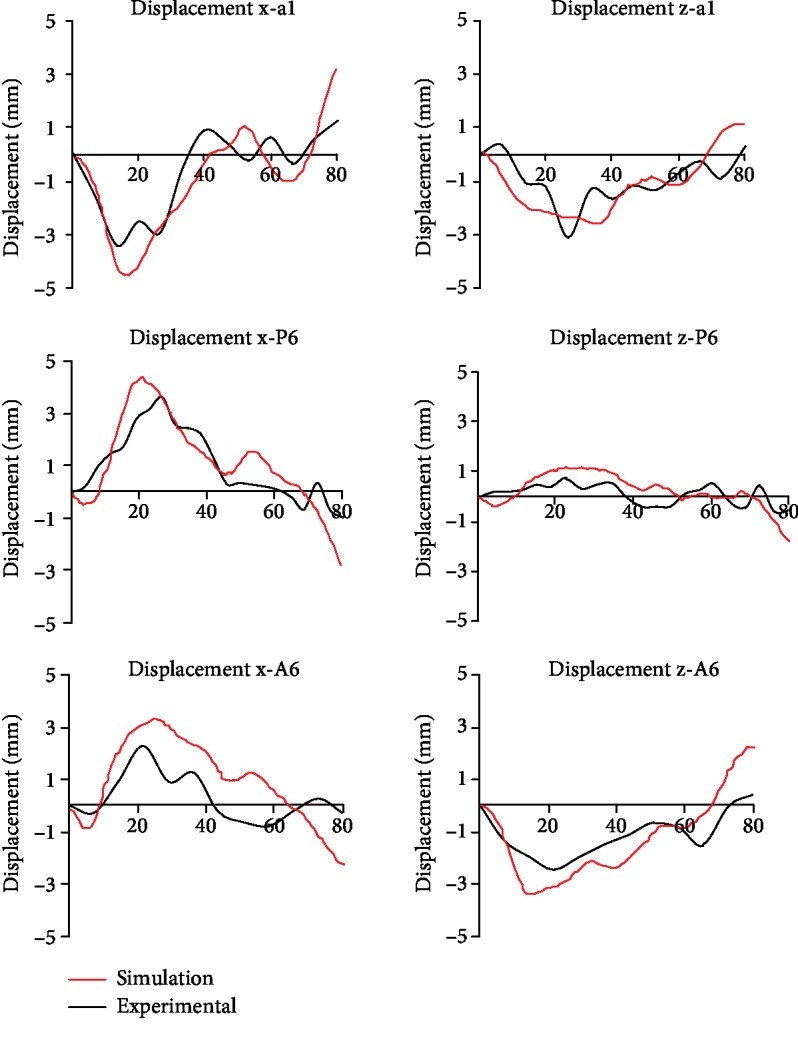
Comparison of relative displacement curves of brain in C383-T3 experiment and simulation.

The brain-skull relative displacements of the simulation and experiment are shown in Figure 5. It can be seen from the figure that the relative displacements of the nodes in the *X* and *Z* directions are basically consistent with the experimental results, and the peak is slightly lower than the experimental results. The difference in results may be due to differences in geometric and material properties between the experimental and finite element models, as well as the deviation in the location of experimental test points.

### 2.3. Research Methods of BHBT

**Figure 6:**
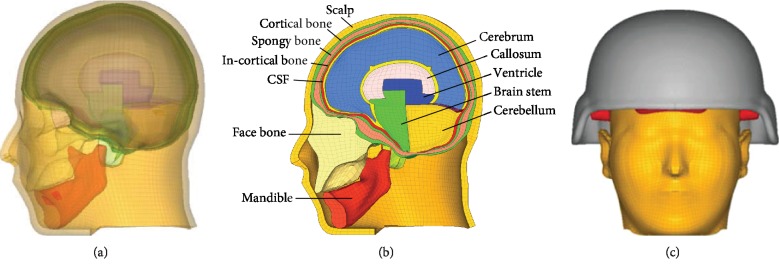
Finite element models.

Because the foam material is soft, after wearing a bullet-proof helmet, the brain will have a certain compression deformation on the initial soft foam. In the simulation, the established foam finite element model is prestressed by HyperMesh in order to simulate the response of the bullet-proof helmet in the real environment. The prestressing process is to fix the head and apply gravity on the helmet; the outside of the foam will be deformed to fit the helmet and the inside of the foam will be deformed to fit the head [28]. The final constructed finite element models with detailed anatomical structure are shown in Figures 6(a) and 6(b); the assembly of the bullet- proof helmet, the human brain, and the foam is shown in Figure 6(c). We define the contact between the helmet and the foam and the foam and the scalp as face-to-face contact.

**Figure 7:**
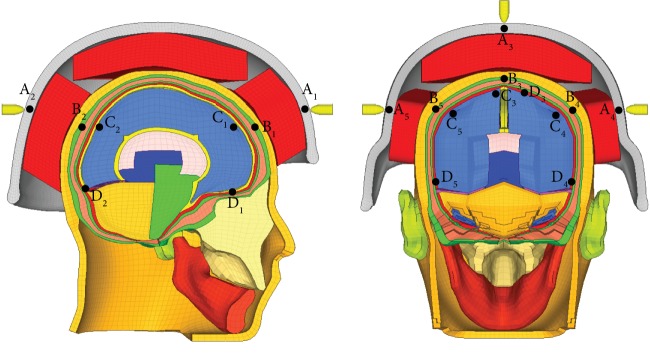
Measurement location of different impact points.

We use the established and verified human brain biomechanical model and the bullet-proof helmet finite element model to study the parameters of brain injury with different impact directions, speeds and structures. We test the location shown in Figure 7 to obtain the amount of deformation on the back of the helmet, the skull stress (at the skull), the absolute displacement of the brain (at the cerebrum), and the intracranial pressure (at the dura mater) and evaluate the damage, corresponding to the test point locations which are shown in Table 2. Since the impact time is extremely short, the fixed problem of the head model is not considered. Firstly, the helmet-cranial model was impacted on the frontal, rear, top, left side, and right side of the bullet-proof helmet at a speed of 400 m/s by the opponent's bullets to study the damage of the brain in different impact directions. Then, the helmet-cranial model was impacted by bullets at 400 m/s, 420 m/s, 440 m/s, and 460 m/s from the right. Also, the helmet-cranial model was impacted by a handgun bullet, fragmentation bullet, and rifle bullet with the same kinetic energy of 360 J on the front of the helmet to simulate the impact of different structures on the brain injury.

## 3. Result

### 3.1. Influence of Different Impact Directions on Helmet Deformation

**Figure 8:**
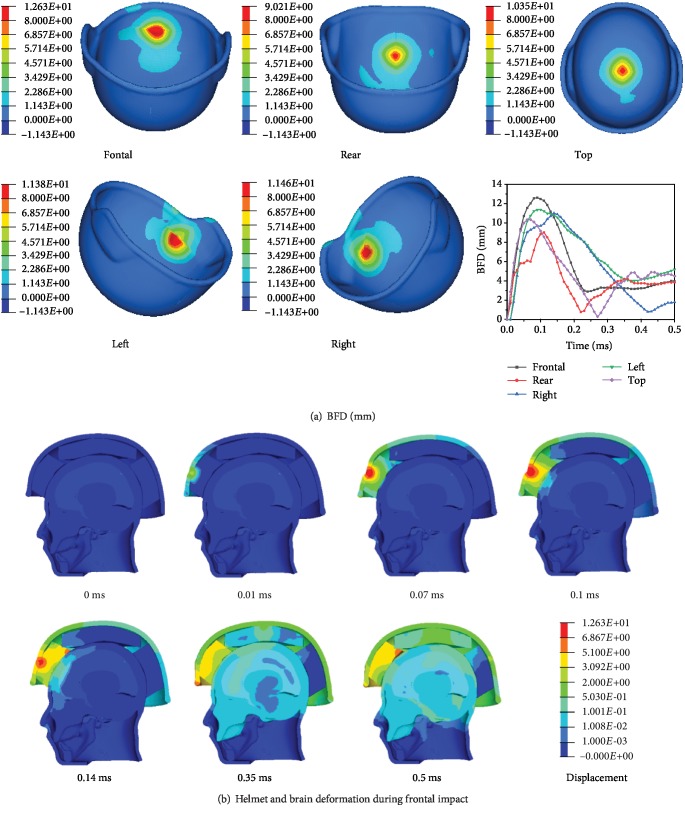
Helmet and brain deformation.

The simulation results of the handgun bullet impacting the bullet-proof helmet from different impact directions at 400 m/s are shown in Figure 8. The damage shape of the helmet is circular when impacted in different directions. As shown in Figure 8(a), the BFD value of the output point A (output point position as shown in Figure 7) is different at different positions, and the BFD of the front impact has the maximum value (12.63 mm at 0.09 ms), and for the top collision (10.35 mm at 0.07 ms), backward collision (9.021 mm at 0.11 ms), right impact (11.46 mm at 0.14 ms), and left impact (11.38 mm at 0.1 ms), the result is basically consistent with the experiments done by Rodríguez-Millán et al. [14]. The trend of the BFD value of different impact directions with time is basically the same. As shown in Figure 8(b), in the change of the model displacement with the time in frontal impact, the bullet contacts the helmet at *t* = 0.01 ms, the helmet begins to deform, and the skull begins to deform at *t* = 0.07 ms, The BFD maximum reach at *t* = 0.09 ms.

### 3.2. Influence of Different Bullet Speeds on Brain Injury

**Figure 9:**
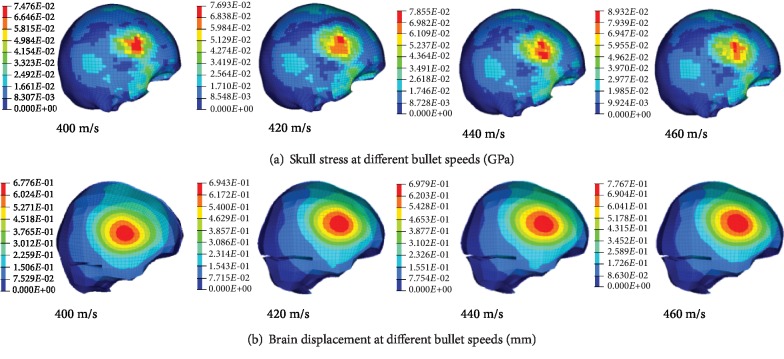
Head response at different bullet speeds.

The simulation results of different bullet speeds impacting the right direction of bullet-proof helmets are shown in Figure 9. The stress cloud map and brain displacements of the cranial skull caused by different bullet speeds impacting bullet-proof helmets are shown in Figure 9. The higher the speed and the greater the BFD value, brain displacement, skull stress, and dura mater pressure, the more serious is the damage of the brain.

### 3.3. Influence of Different Bullet Structures on Brain Injury under the Same Kinetic Energy

**Figure 10:**
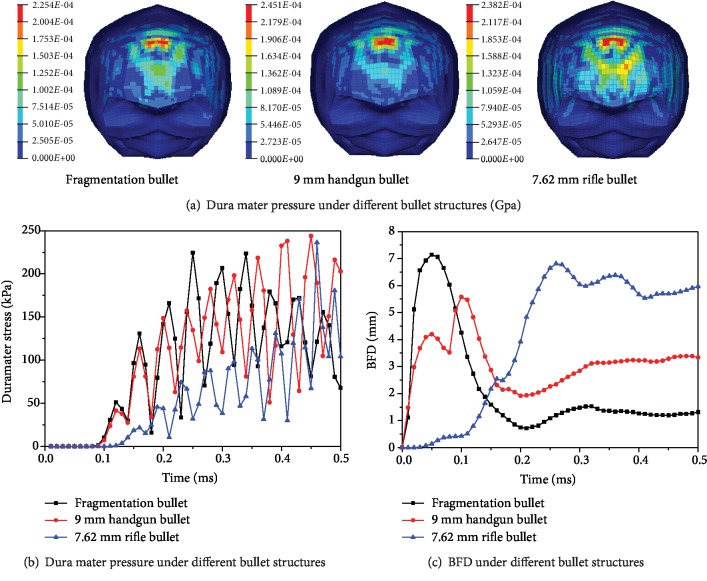
Brain response under different bullet structures.

Under the same 360 J kinetic energy, the speed of the fragmentation is 798 m/s, the speed of the 9 mm handgun bullet is 300 m/s, and the speed of the 7.62 mm rifle is 256 m/s. The simulation results of different bullet structures impacting bullet-proof helmets are shown in Figure 10. The dura mater pressure maps of different bullet structures impacting the bullet-proof helmet are shown in Figure 10(a). The highest dura mater pressure is 245.1 kPa for the 9 mm handgun bullet, 238.2 kPa for the rifle bullet, and 225.4 kPa for the fragmentation bullet. Three kinds of bullets impacting bullet-proof helmets to obtain the dura mater pressure output point D_1_ (output point position as shown in Figure 7) are shown in Figure 10(b). The small mass fragment has the first dura mater pressure change, and the last dura mater pressure change is caused by the largest mass rifle. The BFD values of the output point A_1_ under the three types of bullets impacting bullet-proof helmets are shown in Figure 10(c). When the maximum deformation is reached, the fragmentation bullet is the fastest, the handgun bullet is the second, and the rifle bullet is the slowest. The maximum deformation degree of the helmet is the largest for the fragmentation bullet with 7.1 mm, followed by the rifle bullet with 6.8 mm, and the lowest peak of the handgun bullet is 5.6 mm.

## 4. Discussion

The core question of the BHBT and protective performance of bullet-proof helmets is whether it can propose a protective structure and protection method based on real human brain injury, instead of the parameters and methods based on the currently used dummy brain, sludge, or gelatin. At present, the V50 method [16] can accurately evaluate the penetration resistance of the helmet, but it cannot effectively evaluate the BHBT. Therefore, the focus of research and the primary task are to evaluate the index and criteria of BHBT and then further guide the design of the helmet through a large number of experiments, parameter research, and optimization methods.

In this paper, the high-precision human brain biomechanical model is established. Under the LS-DYNA environment, the BHBT caused by the bullet impacting the helmet with high speed is simulated and verified, and the analysis and parameters are studied. The skull fracture was evaluated by the stress of the skull, and the brain injury was evaluated by brain displacement and dura mater pressure. The simulation results of the brain model under high-speed impact are consistent with the overall trend of Raymond et al. [29]. The results show that the established brain biomechanical model can correctly reflect the biomechanical response of the human brain; it has good sensitivity to brain dynamic response under different loading conditions and different parameters, which can provide reference for the evaluation of BHBT and the optimization design of subsequent helmets.

It can be seen from the parametric study that there is a significant difference in the damage of the bullet from the bullet-proof helmet in different directions. Studies have shown that BHBT is related to BFD [16]. The BFD of frontal, rear, top, right, and left obtained from different impact directions in this paper are basically consistent with Rodríguez-Millán et al. [14] and others. The comparisons are shown in Table 3. With regard to the trend of the BFD value with time in different impact directions and the change of the model displacement with time in the front, Li et al. [16], Rodríguez-Millán et al. [14], and others also reported similar behavior. The reasons for the differences may be differences in the simulation model, differences in geometric and material properties between different samples, and deviation of experimental test points. The human brain biomechanical model used for this paper includes the scalp, hard bone tissue, brain tissue, and soft tissue, which is closer to the real situation of the human brain. The simulation results further prove the accuracy of the model and accurately reflect the biomechanical response of BHBT caused by the bullet impacting the helmet.

**Figure 11:**
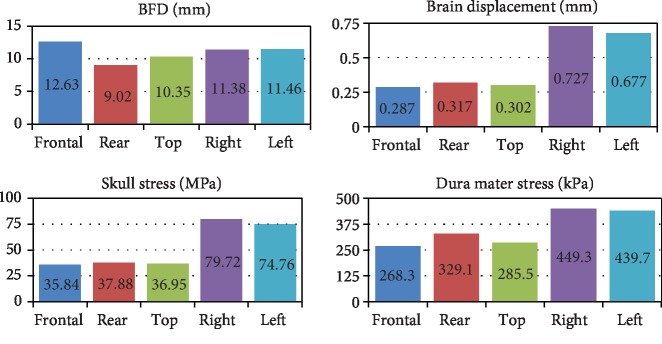
Comparison of peak brain damage in different directions.

The peaks of BHBT from different directions are shown in Figure 11. The brain injury is related to the curvature of the helmet. The larger the deformation of the helmet is, the smaller the brain injury is. The BFD value on the right side is a little larger than that on the left side; the skull stress, brain displacement, and dura mater pressure peaks are smaller than the left. The damage to the brain is also related to the layout of the helmet foam interior. The foam padding area on the left and right sides which consists of two foams with gaps is smaller than that on the other three directions. The simulation results show that the brain displacement and the skull stress are about twice higher when impacted by the left and right sides than when impacted by the front, back, and top.

The injury caused by a high-speed bullet impacting the helmet becomes more serious with the increase of the bullet speed. As the speed increases, the stress of the skull increases and the brain displacement occurs. Rafaels et al. [3] reported that there was no fracture in the low-speed lower brain, a crack at the medium speed, and a fracture at high speed, and there were brain displacements in the seven brain tests. The results of this paper are consistent with the experiment mentioned above.

**Figure 12:**
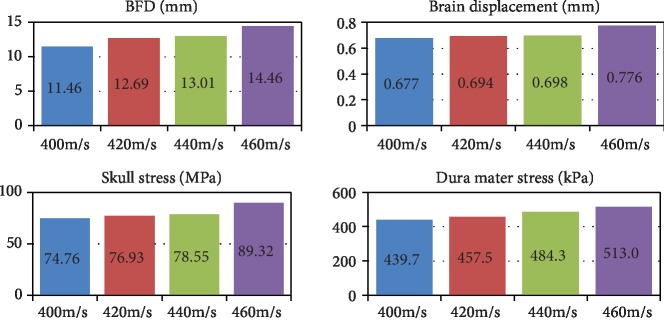
Comparison of peak values impacted by different projectiles.

The peaks of BHBT caused by different bullet velocities impacting bullet-proof helmets are shown in Figure 12. BFD value, skull stress, brain displacement, and peak dura mater pressure can better reflect the brain injury. Simulations show that BFD, skull stress, brain displacement, and dura mater pressure peaks increase with speed.

There are differences in the degree of brain injury caused by different bullet structures impacting bullet-proof helmets under the same kinetic energy. This paper refers to Wang et al. [30] and other people's research on the damage characteristics of landrace which wear on them the body armor with impacts of different structures of rifle bullets. The initial energy of the bullets of three different types of missile structures was adjusted to the same by changing the speed of the bullets. In the same situation as the kinetic energy studied in the literature, different structures are consistent with different brain damage results.

**Figure 13:**
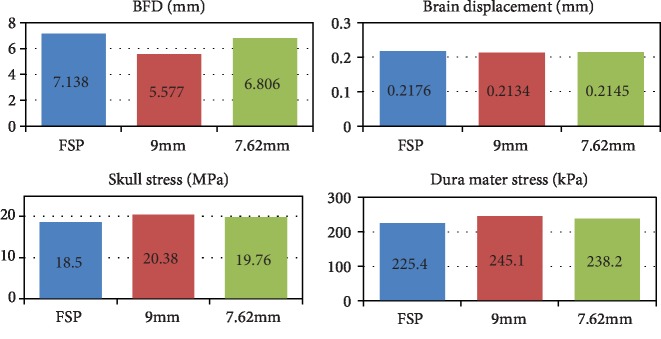
Comparison of peak values impacted by different projectile structures.

The peaks of the BHBT caused by different bullet structures impacting bullet-proof helmets are shown in Figure 13. When the different structures are impacted on the bullet-proof helmet, the energy release mode is different due to the structure of the missile body and the high velocity collision between the projectile and the bullet-proof material; the damage caused to different parts of the brain is inconsistent. The velocity of the fragmentation bullet is the largest, which results in the largest BFD value and the largest brain displacement peak, but the skull stress peak is the smallest. The 9 mm handgun bullet is smoother than the 7.62 mm rifle bullet, which results in the smallest BFD value, the lowest brain displacement, but the largest skull stress peak.

## 5. Conclusion

The results show that the brain model used in this paper can reflect the biomechanical response of the human brain and has sensitivity to brain dynamic response under different impact conditions. The impact position, impact velocity, and structure of the bullet have a significant influence on the skull and brain responses. In particular, the frontal impact of the skull produces the largest amount of BFD, and when the impact directions are from the side, the skull stress is about twice higher than from other directions; as the impact velocity increases, the BFD, brain displacement, skull stress, and dura mater pressure increase, and the brain damage caused by different structural bullet bodies is different under the condition of the same kinetic energy and the skull stress caused by the handgun bullet is the largest. The findings indicate that when a bullet impacts on the bullet-proof helmet, it has a higher probability of causing brain displacement and intracranial high pressure. Intracranial pressure higher than 235 kPa could result in serious brain injury, and the tensile fracture stress for the skull is around 75 MPa [28]. The brain damage caused by different structural bodies is different when the kinetic energy is consistent. Although we have studied the brain injury caused by different bullet speeds, different impact directions, and different bullet structures, there are still many shortcomings to be further studied, such as the impact angle. As mentioned in the article [16], a 45 deg oblique frontal impact leads to a lower head injury risk than a 90 deg frontal impact. In the future, we will continue to study the damage of the brain from different impact angles and so on. This paper can provide reference value for helmet optimization design and antielasticity evaluation and provide the theoretical basis for protection and rescue.

## Figures and Tables

**Table 1:** Bullet material parameters.

Projectile type	*ρ* (kg/m^3^)	*E* (GPa)	*ν*	Yield stress (GPa)	Tangent modulus (GPa)
Handgun bullet [20]	8110	210	0.3	0.792	21
Fragmentation bullet [23]	7830	206.8	0.3		
Rifle bullet armor [24]	8960	124	0.3		
Rifle bullet steel core [24]	7850	206	0.3		

**Table 2:** Corresponding serial number of measurement impact point.

	Frontal	Rear	Top	Right	Left
BFD	A_1_	A_2_	A_3_	A_4_	A_5_
Skull stress	B_1_	B_2_	B_3_	B_4_	B_5_
Brain displacement	C_1_	C_2_	C_3_	C_4_	C_5_
Intracranial pressure	D_1_	D_2_	D_3_	D_4_	D_5_

**Table 3:** BFD values of different impact directions compared with the literature.

	Frontal (mm)	Rear (mm)	Top (mm)	Right (mm)	Left (mm)
Simulation results	12.63	9.021	10.35	11.46	11.38
Experiment in literature [14]	12	9	11	11	6

## Data Availability

The data used to support the findings of this study are available from the corresponding author upon request.
